# The failure to protect the rights and needs of children in Gaza

**DOI:** 10.3389/fpubh.2025.1708975

**Published:** 2026-01-13

**Authors:** Sharon Bessell, Cadhla O'Sullivan

**Affiliations:** Crawford School of Public Policy, College of Law, Governance and Policy, The Australian National University, Canberra, ACT, Australia

**Keywords:** children, human rights, socio-ecological analysis, humanitarian law, war, conflict, Gaza

## Abstract

Over the past 80 years, international human rights and humanitarian law have emphasized the protection of children during war and conflict as one key principles of international humanitarian and human rights law. The assaults on Gaza, following the Hamas attacks on Israel on 7 October 2023, have resulted in shocking violations of children's human rights. Two factors make Gaza an extraordinary example of such breaches of international law. First, the depth and extent of rights violations and the pain and destruction to which children are being subjected. Second, that those violations are playing out in full view of the world through both traditional and social media. In this paper, we provide a narrative review, showing the extent to which children's basic needs and human rights have been violated. We then undertake a socio-ecological analysis of the extent to which socio-ecosystems that can support children have been damaged. We argue that the failure of international action to protect children in Gaza is more egregious because violations have been live-streamed around the world and carried out in full view of a global audience. Moreover, while the violations against children in Gaza represent breaches of those children's human rights and cast doubt over the ability and preparedness of the international community to protect children's human rights anywhere.

## Introduction

From October 2023, following the shocking attacks by Hamas on Israel, the world's attention focused on Gaza as the Israeli Government ordered the systematic destruction of the territory, resulting in loss of life on a mass scale, displacement and starvation. In the first weeks of the attack, United Nations Secretary General Antonio Gutteres (1) gave a dire warning: “Gaza is becoming a graveyard for children”. In September 2025, almost 2 years after the attacks began, the UN Independent International Commission of Inquiry on the Occupied Palestinian Territory (2), found that the Government of Israel was committing genocide against Palestinians in Gaza. In releasing the report, Commission Chair, Navi Pillay, stated “[e]very day of inaction costs lives and erodes the credibility of the international community. All States are under a legal obligation to use all means that are reasonably available to them to stop the genocide in Gaza” (130).

Between October 2023 and August 2025, the violence had escalated resulting in famine in parts of Gaza, as a direct result of blockades by the Israeli Government, and Prime Minister Netanyahu's declaration of his intention to fully occupy large parts of the territory. The Israeli Government's attack on Gaza came after the terrible violence carried out by Hamas against Israel on 7 October 2023, which included the killing of almost 1,200 people and taking of over 240 hostages (3). Among the hostages were 36 children, ten without family members (4). The response of the Israeli Government was one of brutal collective punishment. It is, however, important to recognize that the origins of the most recent conflict, are to be found decades earlier than 2023 and are embedded in a history of violence and dispossession. Byman [(3), p. 61] has argued “both Hamas and Israel may be losing. Each can point to quite real successes against the other, but when the fighting subsides, both are likely to be worse off than they were when the war started.” This may be so, but those who have lost the most are the people of Gaza as assaults from the Israeli military escalated. Children are suffering deeply as they are subjected to egregious human rights abuses and their most fundamental needs are denied. This article focuses on the rights and needs of children in Gaza in the context of the attacks carried out against them from October 2023 until 31 August 2025, when the International Association of Genocide Scholars declared that “Israel's policies and actions in Gaza meet the legal definition of genocide in Article II of the United Nations Convention for the Prevention and Punishment of the Crime of Genocide (135)” and “constitute war crimes and crimes against humanity as defined in international humanitarian law and the Rome Statute of the International Criminal Court” (5).

We put forward three arguments in this paper. First, the psychological and physical harm inflicted on children in Gaza is of such magnitude, it demands immediate international action. Over the past three decades, the evidence for the ways in which war, conflict and violence impact children has expanded and the psychological harms and trauma caused by war are incontrovertible. In Gaza, children are being exposed to both human rights violations and the destruction of their socio-ecosphere. All protective factors have been stripped away.

Second, because the violations of children's human rights and basic needs are playing out in full view of the world through both traditional and social media, the failure of the international community to act to protect children is indefensible. Lack of knowledge or evidence of the extent of violations against children cannot be used as a justification for inaction. There are few gross violations against children that have been so well documented or so powerfully communicated.

Third, while the failure to protect children in Gaza has devastating consequences for those children and their families, it also undermines international norms around the protection of children during conflict. Thus, international action to end ongoing violations of children's human rights is imperative both for the children whose lives, dignity, future and interests are under assault and if human rights—and particularly children's human rights—are to have any meaning globally.

This article begins with a brief overview of the effects of war and conflict on children, highlighting the physical, social and psychological impacts. We map the rights to which children are entitled under international humanitarian and human rights law. We then turn to a narrative review of the human rights violations to which children have been subjected, creating a catalog of the atrocities that have been inflicted. Against the backdrop of violations identified through the narrative review, we draw on Gal (96) and Bronfenbrenner (6–8) to undertake an analysis of children's socio-ecosphere that places human rights violations and denial of basic needs within the everyday systems of children's lifeworlds. We also examine the broader context of the conflict over time and within geopolitical interests. In doing so, we highlight both the damage wrought on individual children and the complete dismantling of every ecosystem of the overall socio-ecosphere within which children live. within which children live.

## The effects of war, conflict and violence on children

Ladd and Cairns [(9), p. 14] observed almost three decades ago that ‘modern wars have become increasingly lethal for civilians', documenting the rise of civilian casualties of armed conflict over the twentieth century. That trend continued in the succeeding decades, with an increasing proportion of civilians—children in particular—experiencing the direct effects of conflict. In 2023, violence against children in armed conflict was reported by the UN as having reached unprecedented levels (10). In 2024, the UN Secretary General's report on children and armed conflict reported that violence against children had increased a further 25% over the previous year. Violations against children in Gaza were highlighted in the report (11).

As the incidence of violence against children in war and conflict has increased, so too has the body of evidence demonstrating the devastating physical, psychological and social impacts that result (12–15). Existing evidence points irrevocably to psychological harm being inflicted on children in Gaza. That harm is occurring within a longer-term context of trauma. As Manzanero and colleagues (16) suggest, the majority of children in Gaza were exposed to war trauma prior to the most recent conflict. Abudayya and colleagues' (17) scoping review of the impacts of conflict on children in Gaza found a high prevalence of PTSD, depression and anxiety.

War-related trauma has both immediate and long-term impacts on children, often manifesting as developmental regression, inconsolability, agitation, and social withdrawal (16, 18). In their research on the ways in which war trauma impact children, Green and Kocijan-Hercigonja [(19), p. 585] succinctly and powerfully document children's direct experiences of “bombing, shelling, sniper fire, and the destruction of their homes and villages”, as well as witnessing the wounding or deaths of loved ones. Green and Kocijan-Hercigonja are describing direct war trauma experienced by children in the war in Bosnia and Croatia in the 1990s. The description is equally applicable to the experiences of children in Gaza today. The nature of the trauma suffered is shaped by children's age and stage of development (20). For example, in Bosnia and Croatia children experienced post-traumatic stress disorder, phobic anxiety, depression and alienation, while adolescents experienced similar responses, but also showed high levels of aggression (19). Begovac and colleagues (21) have highlighted the impacts of war stressors on the self-image and trauma experienced by adolescents.

Notably, while all children living in war zones are affected, just as the degree and nature of the conflict varies so too does the impact on children (22). Studies from Lebanon have indicated that being directly exposed to shelling, death and forced displacement intensifies children's experiences and the level of psychological distress, including regression, depression, and aggression (23). Moreover, trauma created by experiences of war in early childhood has been shown to have lasting effects throughout life (24). Separation from parents and family exacerbates the level of trauma and the stressors experienced by children. While some studies have indicated that separation may play out as prosocial behavior, there are lasting and damaging consequences for children (22). As discussed below, in Gaza all children are directly experiencing exposure to shelling, sniper fire, death and forced displacement. For children being born into the conflict, the trauma begins before birth (25).

Chasson and colleagues' (26) systematic review of the impacts of war-related trauma on early child-parent relationships highlights the association between parents' PTSD symptoms and emotional distress and their relationships with their children. Mothers experiencing PTSD and emotional distress found it difficult to interact with or respond to their children. Moreover, mothers (as well as children) experienced high levels of separation anxiety. While Chasson and colleagues identified some findings of positive parent-child interactions in conflict situations and protective factors, all 22 studies reviewed found adverse relational outcomes due to war-trauma (26).

The literature on the psychological and developmental impacts of war on children highlight the extent to which children's mental health, levels of distress and trauma, and relational health are impacted by war and conflict. Both the physical and psycho-social impacts of war and conflict create lifelong problems. Research has highlighted the ways in which exposure to conflict and large-scale destruction during childhood damages health, education, and labor market outcomes decades after the conflict has ended and throughout adulthood (27). The extensive literature on the impacts of war and conflict on children makes clear the damage to children's lives, fundamental needs and basic human rights, immediately and into the future. The recent attacks on Gaza are among the most severe in recent history (28) and the nature of human rights violations egregious. Before providing a narrative review of those violations, as a basis for our socio-ecological analysis of the dismantling of the systems that generally protect children, we first map the rights to which children are entitled under international human rights and humanitarian law.

## Children's human rights and war

By definition, war has devastating impacts on children, threatening their lives, violating their human rights, and denying their basic needs. Recognition of the vulnerability of children in war and conflict has resulted in the development and endorsement of a range of protection measures for children within international human rights and humanitarian law treaties and conventions. Throughout the twentieth century, efforts to protect children in times of war have been a central concern of international humanitarian law, as the pain and damage inflicted on children has steadily increased (10).

The Geneva Conventions, adopted in the wake of World War Two, represent a critical point in the development of International Humanitarian Law. The fourth Geneva Convention addresses the Protection of Civilians in Times of War, identifying children (generally specified as those aged below 15 years) as entitled to special protections and preferential treatment, including recognition of the importance of family and of care and education. Section III, Article 57 of the Geneva Conventions requires that occupying powers “facilitate the proper working of all institutions devoted to the care and education of children.” Moreover, the Section III states:

The Occupying Power shall not hinder the application of any preferential measures in regard to food, medical care and protection against the effects of war, which may have been adopted prior to the occupation in favour of children under fifteen years, expectant mothers, and mothers of children under seven years (29).

The special protections afforded to children in times of armed conflict are further developed in the 1977 Additional Protocols to the Geneva Conventions of 1949. While particular focus is given to prohibitions on the recruitment of children into conflict, broader issues are addressed (30). Additional Protocol 1 prohibits targeting of the civilian population and individual civilians and extends the Geneva Conventions to all medical personnel (31). Additionally, Protocol II (31) states “children shall be provided with the care and aid they require”, in particular education, reunion with families, protection from hostilities, and temporary removal to safer areas (and to be accompanied by parents or others responsible for their safety and well-being). However, as Dixit (32) observed two decades ago, children continue to be brutally impacted by conflict indirectly and directly, as international humanitarian law is breached. He calls the special protections provided to children to be adhered to in both letter and spirit, and identifies the crucial role for the international community in demanding compliance as a matter of conscience. Dixit's call to action is especially poignant in regard to the assault on Gaza since October 2023, given the number of children killed and maimed, and the failure of the international community to demand compliance with international law (33, 34).

Also adopted in the wake of World War Two, the Universal Declaration of Human Rights (UDHR) provides the foundation of the international human rights system. The preamble of the Declaration sets out the vision of peace and human rights as interrelated.

Whereas disregard and contempt for human rights have resulted in barbarous acts which have outraged the conscience of mankind, and the advent of a world in which human beings shall enjoy freedom of speech and belief and freedom from fear and want has been proclaimed as the highest aspiration of the common people.

The UDHR forms the foundation of the international human rights framework but pays little attention to children specifically. Indeed, only Article 25 of the UDHR refers to children in a meaningful way, guaranteeing special care and assistance to “childhood and motherhood”. As discussed later, the adoption of the United Nations Convention on the Rights of the Child (UNCRC) in 1989 extended human rights explicitly to children.

The UDHR proclaims the “inherent dignity and of the equal and inalienable rights of all members of the human family [as] the foundation of freedom, justice and peace in the world”, but did so at a time when more than one third of the world's people were living under colonial rule. While more than thirty countries gained independence between 1945 and 1960, Palestine's right to exist as a nation state was forcibly challenged, leading to the dispossession of the Palestinian people in 1948 (35).

In 1974, the UN General Assembly adopted the Declaration on the Protection of Women and Children in Emergency and Armed Conflict. While not a legally binding treaty, the Declaration sought to progress the norms of the protection of women and children, enshrined in the Fourth Geneva Declaration, including prohibiting and condemning attacks and bombings on civilian populations.

While children are entitled to special protections under both the Geneva Conventions and the UDHR, it was not until the late 1980s and into the 1990s that children were centerd within international human rights and humanitarian law. Echoing the concern that the rules of law progress as warfare becomes more devastating (36), the greater focus on children in the late twentieth century followed a new pattern of armed conflict that inflicted significant impacts on civilians and communities, and on children (37). Additionally, the UNCRC has prioritized the human rights of children, including in times of war and conflict, within the international human rights framework. The adoption of the UNCRC in 1989 focused global attention on the human rights of children and on gross violations of those rights. The UNCRC incorporates civil, political, social, economic and cultural rights. While the rights enshrined in the Convention are often considered indivisible, the Committee on the Rights of the Child—responsible for overseeing implementation of the Convention—has identified four guiding principles, including the rights to life, survival and development (Article 6).

Article 38 of the UNCRC refers explicitly to the obligation of States Parties to “respect and to ensure respect for rules of international humanitarian law applicable to them in armed conflicts which are relevant to the child” and to ensure the protection and care of children. In practice, these agreements have been regularly violated and the number of children killed and maimed in wars and conflicts around the globe has increased in recent years (10, 11).

In 1993, the Committee on the Rights of the Child recommended a global investigation of the impacts of war and conflict on children. The UN Secretary general appointed Graca Machel, former first lady and Secretary of Education of Mozambique, as the expert lead for the global study, which was undertaken over two years (37). The findings of the study showed “that children face a holistic assault from direct violence and from the structural violence associated with poverty, malnutrition, and inadequate health care” [(37), p. 326]. Following Graca Machel's 1996 report *The Impact of Armed Conflict on Children* the United Nations Security Council has adopted a thematic agenda of Children and Armed Conflict. Koo-Lee (38) has described this agenda as “built upon a single pillar: the protection of children”. The Machel Study (136) marked a watershed in the global attention given to the impacts of conflict on children, leading to the UN Office of the Special Representative of the Secretary-General for Children and Armed Conflict and putting the issue of children and armed conflict on the agenda of the UN Security Council with a series of resolutions to protect children resulting (38).

Despite the many advances made to center the protection of children in war and conflict, in practice, these agreements have been regularly violated and the number of children killed and maimed in wars and conflicts around the globe has increased in recent years (10, 11). Grave violations have been reported in Burkina Faso, the Democratic Republic of Congo, Myanmar, Somalia, Sudan, Syria, Ukraine and Gaza. The assault on Gaza since October 2023 is among the most heinous, ongoing attacks on children in recent history. Moreover, those attacks have received immense and sustained global media coverage, with the destruction of children's lives playing out publicly. While there is rightly debate about the nature of the coverage (39), our argument is that the depth and extent of the coverage means no government can feign ignorance to the destruction of children's lives. The war against Gaza has impacted children in ways that many accounts consider to be unprecedented. The direct targeting of children in Gaza has been well documented, as we detail below. While it is not a new phenomenon, the nature and extent of assaults on children brings a new dimension to ways in which children's human rights are violated and basic needs denied as a result of conflict.

As the overview above indicates, both international human rights and humanitarian law requires that children be protected during war and conflict. Now well-established evidence, discussed in more detail below, indicates that the war on Gaza represents new depths in the disregard of children's human rights, basic needs, and very survival. As we argue below, not only has international law designed to protect children been discarded, the violations that have occurred have been live-streamed globally. The following section provides a narrative review of the violations to which children have been subjected in Gaza since October 2023. While by no means comprehensive, it synthesizes the violations of children's human rights and denial of their most basic needs and highlights the extensive documentation of those violations. Our primary purposes are threefold. First, to present the extent of the trauma children are experiencing. Second, to demonstrate the extent to which those violations have played out in real time in full view of a global audience and the inexcusable lack of action to protect children. And third, to support our argument that the failure to protect children in Gaza has devastating implications for the future of international efforts to protect children whose human rights and survival are under attack. The follow section demonstrates the shocking impacts of children in Gaza and raises serious questions about international commitment to uphold both the letter and spirit of international and human rights law.

## The rights and needs of children in Gaza: a narrative review

In the following sections, against the backdrop of well-established evidence of the impacts of war trauma on children and international commitments to protect children in conflict, we undertake a narrative review of credible reports and documentation to understand the nature and extent of violations of children's human rights in Gaza. Our narrative review is, shaped by the UNCRC within the context of broader human rights and humanitarian law. The purpose of the review is to both identify how children's human rights are being impacted during the 2023 to 2025 attacks on Gaza and to serve as a foundation for the succeeding ecological systems analysis. The narrative review also demonstrates that while children's right to survival was under threat from the first days of the conflict, the depth and nature of violations escalated over time as starvation was used as a weapon of war and full-scale occupation played out in August 2025.

## Methodology

### Identifying reports

We initially conducted a search of documents relating to the situation of children in Gaza since October 2023. For the purpose of the search the following key words were used: children, Gaza, Palestine-Israel conflict, human rights, children's rights. An initial search of academic articles on children in the occupied Palestinian Territories, with a focus on Gaza, was undertaken using google scholar. Reference lists from these articles were then used to identify additional relevant documents, particularly those from UN agencies. A search was also undertaken, using the same key words, for reports from UN agencies and international humanitarian and child organizations. References provided in these reports were then used to expand the search. There was considerable overlap between reports and other documentation from UN sources; the urgency and escalation of the conflict meant that figures varied depending on the time between writing and publication. The United Nations Office for the Coordination of Humanitarian Affairs (UNOCHA) publishes situation update reports weekly and the Gaza Humanitarian Response Update fortnightly; both were included in the narrative review. UNOCHA situation update reports often include both Gaza and the West Bank; for the purposes of this study, we included only Gaza. These updates allowed for the development of a timeline, which highlighted both the depth of violations and escalation over time. Once the initial timeline was established, additional documentation from UN agencies were added, including reports of the Secretary General, the Special Rapporteur on the Situation of Human rights in the Palestinian Territories, and the General Assembly Security Council.

In undertaking the research that underpins this article, we recognize the long-standing nature of the conflict in Gaza and ongoing human rights violations, discussed later. However, for the purposes of the narrative review we took the Hamas attacks on Israel on 7 October 2023 as our starting point, with an end date of 30 August 2025.

### Determining legitimate/credible sources

In undertaking this review, we acknowledge the challenges of identifying accurate and unbiased sources in an environment of conflict. Throughout this article, we have drawn on peer reviewed research wherever possible; however, the immediacy of events requires a broader focus. We have also relied on expert reports from credible sources, including UN agencies and reputable media outlets. We recognize that the Government of Israel has rejected UN reports and challenged evidence provided, including the numbers of deaths. In 2024, as one example, there was debate around the data used by the UN to determine the number of children and women killed, with claims that the data used were unreliable and politically motivated (40), fuelling debate about the credibility of sources. Jacob (41) suggests that such debates seek to use the scale of atrocities against children to advance ideological and political agendas and are not ultimately useful in the pursuit of justice. We agree with Jacob, while also recognizing both that the number of children killed matters greatly to justice and that those numbers will necessarily be contested in highly charged situations. We also recognize that ensuring the accuracy of data is necessarily challenging in contexts of war, mass civilian casualties, and widespread destruction. In the context of the war on Gaza, all evidence demonstrates the enormity of the loss of life, with many thousands of children killed. Moreover, largely ideologically driven debates over precise numbers are less important in pursuing justice than recognizing children as holders of human rights, documenting the patterns and nature of violations, holding to account perpetrators of rights violations, and creating security for all children. Justice also requires that the systems and policies that enact war are uncovered and challenged (41, 42). The aim of this narrative review is to synthesize what is known about the situation of children in Gaza. We focus not on debates over the numbers of children experiencing atrocities and violations of their human rights (although we do quote credible sources), but seek to highlight the patterns, nature and depth of violations, the impacts on children, and the escalation over time. In doing so, we expose the extent to which the international community and global and national leaders have been aware of the devastating impacts of the conflict on children since the commencement of the war on Gaza.

We drew on the principles of currency, relevance, authorship, accuracy, and purpose to inform our search (43, 44), adding two principles as part of our methodological innovation to ensure credibility of sources: cross-checking and process. We define credible sources as those which are peer reviewed; provided by reliable expert bodies; and/or seek to provide information rather than take a politically motivated position. The UN's mandate is to “take the position of reporting on violations to human rights, bringing it to the world's attention to prevent further abuses occurring and deter others arising” (45). Further, the UN is mandated to provide “information management services to the humanitarian community to inform a rapid, effective and principled response. It gathers, shares and uses data and information, underpinning coordination, decision-making and advocacy” and is grounded in the belief of advocating “for effective and principled humanitarian action by all, for all” (45). Thus, we define UN documents as credible and legitimate. The value of UN reports is considerable, particularly as the blockade of Gaza by Israeli forces prevented entry by research, advocacy, humanitarian and media organizations. Table 1 provides the way in which we applied principles of currency, cross-checking, relevance, authorship, accuracy, purpose, and process to sources, particularly those produced by UN agencies.

**Table 1:** Method for assessing sources.

**Principle**	**Description**	**Reasoning/justification**
Currency	Assess the timeliness of information.	Immediacy of events meant we chose UNOCHA situation updates to inform our search. We searched humanitarian updates for the Gaza strip that were published weekly. For the purpose of this search, we gathered information for each month started October 2023 to August 2025.
Cross-checking	Check veracity against other sources.	Wherever possible, UN documents were crosschecked with academic, peer-reviewed sources to enhance credibility and reliability
Relevance	Determine suitability for the research topic, argument and analysis	The UN provide reporting on violations to human rights in Gaza in a timely and publicly available manner.
Authority	Evaluate the credibility and expertise of the author or organization.	The UN is deemed a credible international organization, assessed against these principles.
Accuracy	Check if the information is verifiable and free from bias	The UN is a reliable expert body; and seeks to provide information rather than take a politically motivated position. Cross-checking supported this.
Purpose	Consider the intent of the source, whether it aims to inform, persuade or entertain	The UN mandate is to inform to an international audience and protect civilians, particularly children, during conflict.
Process	Exclude sources that are not credible, timely or relevant	Having justified UN documents as credible and reliable sources, we set a timeline of October 2023-August 2025. The OCHA situation updates were used to inform the basis of our narrative review and were supported by peer-reviewed journal articles. Key search terms: children, Gaza, Palestine-Israel conflict, human rights, children's rights.

## Findings: the extent of violations of children's human rights in Gaza

We undertake the narrative review and associated analysis through a human rights lens, drawing primarily on the rights guaranteed to all children in the UNCRC. In doing so, we note that Article 2 entitles children to human rights “irrespective of the child's or his or her parent's or legal guardian's race, color, sex, language, religion, political or other opinion, national, ethnic or social origin, property, disability, birth or other status”. As a result of the attacks on Gaza, every right guaranteed under the UNCRC has been violated, including rights to identity (Article 8) and to culture (Article 30), to health and healthcare services (Article 24) and to education (Article 28). While much of the following analysis focuses on violations of the rights to life, survival and development (Article 6 of the UNCRC), we draw out the interrelatedness of children's human rights and the extent to which every aspect of children's lifeworlds has been attacked. Article 6 of the UNCRC states:

1. States Parties recognize that every child has the inherent right to life.
2. States Parties shall ensure to the maximum extent possible the survival and development of the child (137).

Our analysis demonstrates that over an extended period from October 2023, the depth and extent of violence escalated and as did the depth and breadth of rights violations. In the first months of the attack, the human rights of children as individuals were violated. As the conflict escalated, the delivery of food and other essential supplies was blocked, there was widespread displacement of the civilian population, and then full-scale occupation of Gaza City. As a consequence, the rights of Palestinians collectively came under dire threat. Violations of children's rights to survival and to identity led to the argument that the conditions for genocide were in place from early in the conflict (46). This escalation and the devastating impacts for children was well documented and played out on in full view of global leaders.

## Rights to survival, development, health and healthcare

While war threatens the right to survival for everyone directly impacted regardless of age, the impacts on children are especially egregious, as discussed above. Children's right to survival (Article 6 of the UNCRC) is closely associated with rights to development (Article 6 of the UNCRC) and to healthcare and the highest attainable standard of health (Article 24 of the UNCRC). The impact of conflict on children's rights to development and to health are well documented, with violations resulting in immediate and long-term impacts [see also (47–49)]. From the beginning of the assault on Gaza from October 2023, children's rights to survival, development, health and healthcare have been violated, seemingly with impunity. It is important to recognize, however, that children's rights to health and survival were already compromised by decades of occupation, conflict, inadequate infrastructure, and a sixteen-year blockade (50–53). As a consequence, the malnutrition, diseases, infections and injuries created by the bombardment of Gaza from October 2023 have not only created direct threats to children's survival but exacerbated the existing situation characterized by human rights violations.

In December 2023, 10 weeks after the commencement of attacks on Gaza, UNICEF described the devastating impacts on children. Hospitals were quickly overwhelmed as children suffered terrible injuries, including more than 1,000 children reportedly losing one or both legs in the first months of the conflict. Subsequent reporting indicated that each day more than ten children were losing limbs, either through the direct impacts of bombardment or through amputations necessary to save children's lives after severe injury (54). As early in the conflict as February 2024, medical practitioners reported that they were running out of critical supplies, including antibiotics and anesthetic or pain relief. Amputations were being undertaken and severe wounds treated without anesthetic or the medications needed to prevent infection (55). Al Shami and Nashwan (54) highlight the severe emotional and social consequences of amputation for children, and for their families where they are still alive. Amputation has immediate life-threatening impacts and longer-term consequences for human dignity and the ability to function (56). These impacts are exacerbated by the lack of specialized services in Gaza, the impacts of the blockade imposed by the Israeli Government and the associated barriers to medical care related to restrictions on movement and lack of transportation (54). In October 2024, a year after the commencement of the conflict, UNICEF described the impacts of the conflict on children:

In Gaza, at least 14,000 children have reportedly been killed, many more injured, while thousands are likely under the rubble, and an estimated 17,000 others are unaccompanied or separated from their caregivers. More than 55 displacement orders remain in effect, covering up to 86% of the Gaza Strip. Children have lost access to quality healthcare, education, and other services. All children are now in need of mental health and psychosocial support. One year into the war, children's most basic needs remain unmet. Persistent restrictions on the entry of humanitarian aid and commercial commodities, and the inability of humanitarians to safely reach all children and families, have rendered the Gaza population acutely food insecure. All 335,000 children under five are at high risk of malnutrition (57).

The impacts of Israeli military attacks on children in Gaza must be understood in the context of the destruction of essential infrastructure, including direct targeting of hospitals and health care facilities (58). According to the World Health Organization (59) there were 697 attacks on health care in Gaza between October 2023 and May 2025. By May 2025, 94% of hospitals were damaged or destroyed, with only 19 of Gaza's 36 hospitals operational. Describing the destruction of hospitals as “systematic”, the World Health Organization highlighted the courage of health workers who continued to deliver urgent care “amid constant fear and insecurity” (59, 104). The implications for children's right to survival and development (Article 6 of the UNCRC), and to health and heath care (Article 24 of the UNCRC) are catastrophic; and provides important context for the review of human rights violations that follows. By late 2023, the hospital system in Gaza was on the brink of collapse. Over the subsequent year and a half attacks on medical facilities have continued, with detailed documentation of the impacts on children but no action to bring the bombardment to an end (14, 59–61).

## The destruction of infrastructure and the targeting of children

The destruction of infrastructure has resulted in the spread of disease and diarrhea among children, as vaccination programs were severely disrupted (50). Those aged under 2 years were identified as being especially susceptible to malnutrition and death, as breast feeding and age-appropriate feeding became impossible. Young children's health and survival was compromised before October 2023, with preexisting high rates of formula feeding and associated risks of contamination due to unsafe drinking water (62). Since October 2023, the risks to children have increased in severity and scope. The dismantling of WASH facilities as a result of attacks on infrastructure has exacerbated rates of waterborne infections and diseases, including diarrhea and hepatitis A (59, 62–64, 104). In July 2024, Poliovirus type 2 was detected in Gaza for the first time in quarter of a century (65). The World Health Organization and UNICEF responded with a mass vaccination campaign, but ongoing attacks hindered access to some parts of Gaza (59), creating ongoing concerns of a widespread polio outbreak with devastating effects (60). Harghandiwal [(62), p. 8] highlights the threat diarrhea poses to children's survival when they are already experiencing malnutrition. As the war on Gaza has unfolded, disease and infection have threatened children's survival and violated their rights to development, healthcare and the highest attainable standard of health. The impacts on children have been deepened due to attacks on hospitals and the unavailability of both ongoing healthcare and emergency care.

The killing and maiming of children in Gaza constitutes grave violations of children's human rights. In the first months of the attacks, human rights organizations warned that the use of large bombs was resulting in shockingly high numbers of civilian casualties (131). Geospatial analysis of the proximity of bombing attacks with 2,000 lb M-84 bombs to hospitals in the first six weeks of the Israeli military attacks on Gaza indicated “indiscriminate bombing in close proximity to hospital infrastructure” (66). Further concerns were raised about the use of the of white phosphorus (67) to which children are especially susceptible (50). As attacks on Gaza have continued, there has been growing concern that the violation of children's right to survival is due to intentional action on the part of the Israeli government and military, as children are directly targeted (68). The density of Gaza's population and the high proportion of children (almost 50%) mean that heavy bombardment almost inevitably causes mass child casualties. Moreover, there are indications of children being targeted by sniper fire. Palestinian and international medical workers have described treating children with wounds that appear to be the result of targeted sniper fire (33, 69). Medical practitioners have reported treating children with direct bullet wounds to the head and chest, suggesting deliberate targeting, claims rejected by the Israeli defense force (33).

Attacks on hospitals and medical centers have also raised grave concerns that children, and civilian populations broadly, have been targeted. Early in the conflict, Israeli airstrikes on the Al-Nasr Medical Center in Gaza City in November 2023 were described by UNICEF and humanitarian organizations as a direct attack on children, resulting in the deaths of babies when oxygen to the neonatal intensive care unit was cut off (70). The airstrikes on Al-Nasr Medical Center are not an aberration but reflecting the pattern of attacks on hospitals and medical centers that has continued.

## Starvation and the denial of children's right to survival

The attacks on Gaza have led to soil contamination and bio-diversity loss, with devastating implications for agricultural production (71). Access to water, both for drinking and daily use and for agriculture, has been severely restricted (72). The resulting decline in agricultural production created severe food insecurity and greater reliance of external aid.

From March 2025, an already catastrophic situation further deteriorated as the Israeli government suspended the entry of aid into Gaza and privatized humanitarian aid delivery through the establishment of the Gaza Humanitarian Fund (GHF). This signaled a horrifying new dimension to the violation of children's human rights. Reputable humanitarian organizations, including UN agencies, were sidelined and all food aid was directed through GHF, which failed to meet criteria required for humanitarian assistance. As a consequence, access to food became increasingly erratic, inadequate and dangerous. Reports emerged of people on the brink of starvation needing to walk for hours to access GHF food sites and to risk their lives from gunfire around distribution sites (73). Children were actively denied food, leading to widespread hunger, starvation and death and were fired upon when seeking food. By August 2025, parts of the Gaza strip were in famine (74).

Food security and adequate nutrition are among the most fundamental needs of children. The impacts of malnutrition are well-established, resulting in children's physical and cognitive development being undermined and their long-term health compromised (75). Chronic malnutrition is not new for children in Gaza, but has been a constant feature of childhood under occupation. In 2002, a study funded by the United States' Agency for International Development found 13.3% of children to be acutely malnourished, describing the situation as a humanitarian emergency [(76), p. 1460]. A 2004 World Food Program report found over one million Palestinian children – living in both Gaza and the West Bank – experienced food insecurity, with almost that number again at risk (76). However, since October 2023 starvation was used as a deliberate weapon of war, leading to widespread suffering and death.

In mid-2024, United Nations Special Rapporteurs reported that the conflict was leading to famine in Gaza, with increasing numbers of malnutrition- and dehydration-related deaths among children. The group described “intentional and targeted starvation campaign against the Palestinian people…” (77). Significantly, they stated “The whole world should have intervened earlier to stop Israel's genocidal starvation campaign and prevented these deaths”, describing “inaction as complicity” (77). Between May and July 2024, malnutrition among children in northern Gaza was reported as increasing by 300% (77), as the World Food Programme warned of a humanitarian catastrophe unfolding and famine a serious risk (78). The situation worsened into 2025, as starvation became widespread.

The Integrated Food Security Phase Classification (IPC) is a global initiative that monitors and seeks to redress situations of severe food insecurity and malnutrition. The IPC has a five-stage food insecurity classification: (1) Minimal/None, (2) Stressed, (3) Crisis, (4) Emergency, (5) Catastrophe/Famine, with identified interventions for each. In May 2025, the IPC Partnership classified all of Gaza as being at IPC phase 4 and issued a dire warning:

Goods indispensable for people's survival are either depleted or expected to run out in the coming weeks. The entire population is facing high levels of acute food insecurity, with half a million people (one in five) facing starvation (74).

Children were identified as at risk of or experiencing acute malnutrition, with 75% consuming considerably less than the minimum dietary requirements for growth and development. The IPC Partnership estimated that in the year from April 2025 70,500 children under the age of five would experience acute malnutrition without immediate intervention, with 20% of those cases (14,100 children aged between 6 months and 5 years) predicted to experience severe acute malnutrition (74). Severe acute malnutrition is directly associated with child mortality, particularly when children are suffering from comorbidities (79), as is the case in Gaza. Treating severe acute malnutrition in babies under 6 months is especially problematic (80). Children experiencing severe acute malnutrition in the first 1,000 days of life suffer irreversible long-term consequences, including stunting, impaired cognitive development, and increased risk of chronic diseases (79). The IPC Partnership (132) has also identified a significant proportion of pregnant and breastfeeding women as experiencing acute malnutrition. This has immediate consequences for mother and baby, including risk of pre-eclampsia, maternal mortality, hemorrhage and amenia among mothers and low birth weight, pre-term birth, and neonatal mortality among babies (81). Moreover, women who experience malnutrition during childhood are more likely to experience complicated deliveries and have babies with low birth weight (79). In August 2025, the Famine Review Committee declared famine in Gaza Governorate, while Deir al-Balah and Khan Youis continued to experience emergency, indicating that famine is plausible in those areas. The Famine Review Committee [(74), p. 2] called for immediate action:

As this Famine is entirely man-made, it can be halted and reversed. The time for debate and hesitation has passed, starvation is present and is rapidly spreading. There should be no doubt in anyone's mind that an immediate, at-scale response is needed. Any further delay—even by days—will result in a totally unacceptable escalation of Famine-related mortality.

## Violations of the right to education

While children's human right to survival is intimately bound up with rights to health and healthcare, development is closely associated with the right to education. While survival becomes paramount during conflict, children's human right to education should not negated. Schools have the possibility of providing protective environments for children during war and conflict and supporting the wellbeing and mental health of children, teachers and parents (82, 83). Schools have also been identified as providing safe places for children to access services and experience aspects of childhood that are destroyed by conflict (83). Given the overwhelming scale of the destruction of infrastructure and loss of life in Gaza, the potential for schools to offer safe places is limited. Schools have been used as emergency shelters and have come under heavy attack.

In October 2023, the United Nations Office for the Coordination of Humanitarian Affairs (OCHA) (84) reported that eighteen schools operated by UNRWA had been attacked, two of these were operating as emergency shelters for people who were displaced early in the crisis. A further 70 schools operated by the Palestinian Authority were destroyed in successive days of bombing. As a result, in the first weeks of the conflict OCHA (84) reported that more than 600,000 children had no access to education. By the end of 2024, almost all school buildings in Gaza were destroyed or damaged (85). Over 95% of Gaza's 564 schools were reported to have suffered damage, with 88% unusable without significant reconstruction (86). In their 2024 review of education in Gaza and the West Bank, Relief Web (86) reported that “all children in the Gaza Strip [are now] denied access to formal education and the essential protective support it provides”.

The destruction of schools not only violates children's right to education now, it also has long term implications. UNICEF (57) estimated that children's education will be set back by up to 5 years. The UN Secretary General, in his January 2025 report, warned of a lost generation, as children are denied education and subjected to continuous violence.

## Right to identity and culture

David Moshman [(87), p. (88)] has written ‘at the heart of any genocide … is identity', arguing:

The acts of destruction may be aimed at individuals, but the individuals are targeted on the basis of their actual or perceived association with a national, ethnic, racial, religious, political, socioeconomic, or other abstractly defined group. The group must be deliberately targeted …

The UNCRC requires the protection of a child's identity, “including nationality, name and family relations”, as recognized by law without unlawful interference' (Article 8). Alongside Article 8, Article 30 of the UNCRC protects the child's right to “his or her own culture, to profess and practice his or her own religion, or to use his or her own language”. In the context of Gaza, the right of individual children to their identity is being violated as the collective identity of the Palestinian people is targeted. For individual children, the violation of their right to their identity is enacted as the separation of children from their parents and families. Almost the entire population has been displaced and, as early in the conflict as February 2024, UNICEF estimated that 17,000 children have been separated from their families. A new term has entered the emergency medical lexicon: WCNSF—“Wounded Child with No Surviving Family” (89). Accounts have emerged of children writing their names on their own bodies and those of their friends, to enable their identity to be known should they be killed. UNICEF provides young children with name bands to enable them to be identified should they be separated from their families or be killed (90).

The scale of death and destruction also threatens the collective identity and future survival of the Palestinian people. The violations of children's human rights in particular, as synthesized above, point to systematic efforts on the part of the Israeli Government to eradicate Palestinian identity. Moshman [(87), p. 122] has described in other contexts the dehumanization of groups, noting that “not even the complete absence of any political consciousness, as in the case of young children, was relevant”. The power of Moshman's (87) analysis is apparent in the public statements of some Israeli political leaders. An especially chilling example is the public comments of leader of the Zionist Zehut party, Moshe Feiglin, in May 2025: “Every child, every baby in Gaza is an enemy … not a single Gazan child will be left” (91).

Attacks on fertility clinics and maternity hospitals, together with widespread gender-based violence, were identified by the Independent International Commission of Inquiry on the Occupied Palestinian Territory (92) in March 2025 as breaching international law and constituting crimes against humanity. On 26 January 2024, the International Court of Justice “issued a provisional measures order that found “a real and imminent risk” that Israel is in violation of the rights of Palestinians in Gaza under the Genocide Convention” (34). Malhotra and El-Shaarawi [(46), p. 2] argue that genocide poses a threat to “a people's capacity for collective survival” with the aim of not only eradicating “a people's present (and presence), but to annihilate and foreclose their futures”. Since that time, the assaults on collective identity, as well as the individual human rights of children, have escalated. In early August 2025, Israeli Prime Minister Benjamin Netanyahu, despite reported misgivings from senior military officers, announced the intention to fully occupy Gaza and displace the population (133). On 20 August 2025, the UN Office of the High Commissioner for Human Rights (OHCHR) reported mass killings of civilians, further destruction of infrastructure, and attacks on displaced people sheltering in tents and schools. OHCHR (93) issued the following statement:

Considering the imminent risk of the further commission of serious violations of international humanitarian law, States parties to the Geneva Conventions are under an urgent obligation to exert maximum pressure on Israel to immediately halt this offensive, which risks triggering an unprecedented, life-threatening humanitarian crisis and permanently extinguishing the Palestinian presence in Gaza's largest urban area.

As the attacks on Gaza unfolded, not only were the human rights of every child in the territory under immediate threat, so too was the identity and collective future of the Palestinian people.

## Summary findings of narrative review

By July 2025, UNRWA (94) reported that 17,121 children had lost their lives. In total, over 55,000 people of all ages were reported to have been killed in the period October 2023 to July 2025. As noted earlier, while specific numbers have been contested by the Israeli government, there is clear evidence that many thousands of civilians have been killed. Thousands of children have lost their lives and many thousands more have been maimed, lost family members, or been exposed to extreme violence. Hunger and starvation are widespread, with famine declared in parts of the Gaza Strip in August 2025. Ongoing mass destruction and threats from Israeli politicians, including the Prime Minister, that Palestinians would be removed from Gaza threatened children's survival and their right to identity (Article 8 of the UNCRC) and violate the Fourth Geneva Convention (95).

Children's human rights to life, survival and development enshrined in the UNCRC have been systematically violated, as have rights to health care and education and fundamental principles of international humanitarian law. These violations have been well-documented by international agencies and the media, with UN agencies and others repeatedly calling for international action, noting that “international norms were established precisely to prevent such horrors” (93).

This narrative review has synthesized the credible information that documents the ongoing violations of children's human rights and denial of their basic needs in Gaza. It is not comprehensive but powerfully supports the argument that the devastating impacts on children are well known. It also highlights the escalation of violations: starvation is being systematically used against children and the entire population of Gaza, infrastructure has been almost completely destroyed, with hospitals and medical centers targeted, there has been a near-total destruction of agricultural land and complete dispossession threatened. As a consequence, every article of the UNCRC had been systematically violated and every aspect of children's lives damaged or destroyed. From this review, we now turn to a bio-ecological analysis of what these egregious violations mean for children now and into the future. In doing so, we highlight the ways in which children's ecosystems have been systematically dismantled and point toward the depth and breadth of reconstruction that will be needed.

## Discussion and analysis: the dismantling of children's ecosystems

Cataloging the violations of children's human rights in Gaza through a narrative review reveals not only that those violations have been well documented by UN agencies and others, but that they have been livestreamed to the world. The concept of human rights, however, can be abstract, particularly in contexts of war and egregious violations. A socio-ecological analysis is helpful in situating children's human rights within broader systems and uncovering how those systems facilitate or fail to deliver children's human rights and basic needs. A socioecological analysis illuminates the enormous challenge of rebuilding children's lives and moving toward any form of justice, given the level of brutality. It also highlights the moral obligation on international actors that have failed to act as genocide has unfolded.

**Figure 1:**
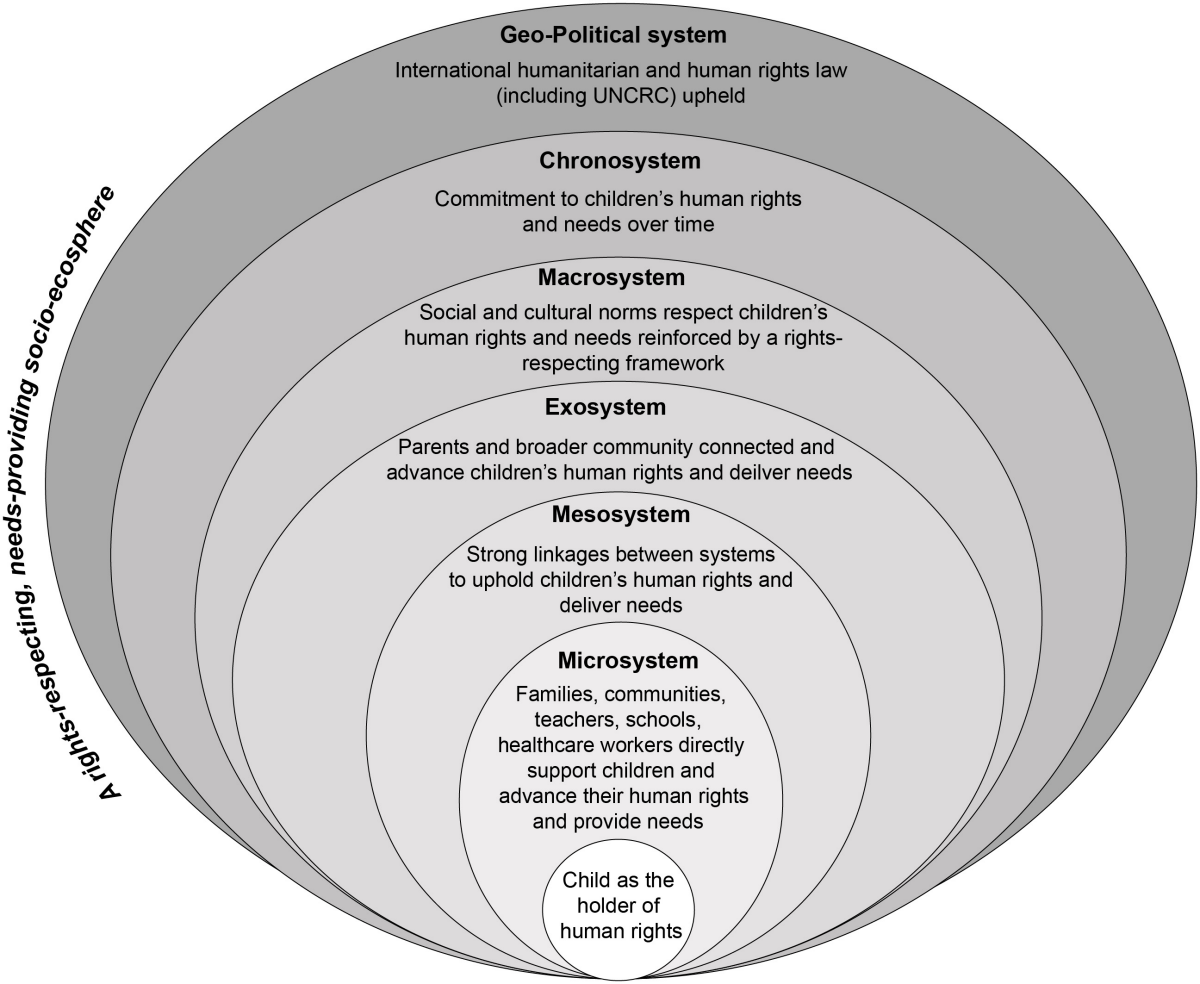
A rights-respecting, needs-provoding socio-ecosphere.

In introducing a needs-rights approach, Gal (2) draws on a socioecological model of child development to demonstrate the ways in which children's physical and psychological wellbeing, and respect for their human rights, are shaped by five concentric circles. The microsystem (such as parents and family); the mesosystem (direct supports outside immediate relationships); the exosystem (systems indirectly impacting children through their families, such as parents work and social connections); the macrosystem (broader cultural and social values); and the chronosystem (temporal experiences which shape the childhood of specific generations, for example, experiences of war and crises) (8, 96). We introduce the concept of an overarching socio-ecosphere, within which ecosystems are nested. In a context of peace, in a rights-respecting and child inclusive society, each socio-ecosystem within the broader socio-ecosphere upholds and advances children's human rights. Such a socio-ecosphere is provided in Figure 1.

In his conceptualization of an ecological model of child development, Bronfenbrenner (8) highlighted the nested, or inter-related, nature of each of the systems that shape children's lives and development. When used in conjunction with the literature on the impacts of war on children, a needs-rights model demonstrates the extent to which direct exposure to conflict damages every aspect of children's life worlds. As demonstrated by the narrative review, the depth and extent of violence and devastation experienced by children in Gaza has resulted in children's basic human rights being denied and a narrowing of their worlds to merely survival. Moreover, this has occurred before an international audience, with details of children's suffering graphically portrayed not only through reports and accounts of aid and humanitarian agencies, but through traditional and social media. In the following sections, we analyse the ways in which children's ecosystems have unraveled since October 2023, exacerbating the trauma being experienced. We also use a socioecological approach to highlight the importance of both the chronosystem (time) and geostrategic/geopolitical systems. We argue that the ways in which violations of children's human rights have played out across each level of children's ecosystems has devastated their lives and lifeworlds and those of future generations. While the catalog of violations exposes the horrors inflicted on individual children, a socioecological analysis highlights the systemic and intergenerational damage that is resulting.

## The devastation of children's socio-ecosphere during the war on Gaza

In this section, we draw on a socioecological model to analyse the impacts of the attacks on Gaza across children's microsystem, mesosystem, exosystem and macrosystem. We then turn to consideration of two additional systems, the chronosystem and the geopolitical system-geostrategic system.

As revealed by the narrative review of rights violations, the systems that support children and their families have been destroyed since October 2023. It is also important to recognize that ongoing conflict had disrupted Gazan children's ecosystems long before that date, as discussed below in relation to the chronosystem, but October 2023 marked the beginning of an unprecedented acceleration of the destruction (97).

## Assault on children's micro-system

The microsystem is characterized by those elements that are closest to the child: family, home, school, and friends, as examples (98). As discussed earlier, the negative impacts of war, conflict and related trauma on children are well established, impacts that are worsened when parents are also experiencing deep trauma and mental ill-health (22, 26). The microsystem is both a crucial protective factor for children in war and conflict, but one that is under immediate threat when civilians are directly targeted. The narrative analysis demonstrates the extent to which children's microsystem has been impacted as a result of mass killing, including of children's family members and friends; loss of their homes, schools and communities; and for many thousands, separation from their families since October 2023. It is also important to highlight that the micro-system surrounding children in Gaza was fragile prior to October 2023. Significantly, however, earlier studies of children's mental health and wellbeing in Gaza have highlighted the ways in which children were supported by parents, family members and teachers (99, 100). In a 2018 study of children's mental health during war, Diab et al. (99) identified the association of child psychological distress among children living in a refugee camp in Gaza with everyday stressors, such as poverty, lack of resources and family conflict. Diab et al. [(99), p. 212] found “a significant positive correlation between (severe war trauma and more mundane stressful events), indicating that exposure to loss, destruction, and atrocities often results in socioeconomic hardships in the context of long-lasting unresolved military conflict.”

In understanding how the conflict impacted the microsystem surrounding children, it is important to recognize that every element of that system has been dismantled. Long-term conflict had already created a context of poverty, displacement, poor infrastructure, and land confiscation creating significant risks to children's mental health and quality of life (101). Those stressors have been magnified though starvation; massive destruction of homes, schools, and hospitals; displacement of over 90% of the population (102), and, as of August 2025, the Israeli government's announcement of full occupation of Gaza. In this context, the protective factors of family and school are stripped away.

## The targeted unraveling of the mesosystem and exosystems

The mesosystem connects children's microsystem to broader systems that children do not encounter daily but nevertheless shape their lifeworlds. For example, schools connect children to education policies within the macrosystem. As discussed in the narrative review, schools have the possibility of acting as protective and normalizing environments for children in contexts of conflict. The destruction of schools represents the unraveling of a key element of the mesosystem. Other crucial elements of children's mesosystem have been destroyed through direct attacks on hospitals and healthcare centers, which we documented in the narrative review. International humanitarian efforts to fill the void created by the dismantling of hospitals and schools have been derailed by attacks on humanitarian workers (103), blockages on the entry into Gaza of essential supplies (104), and the privatization of humanitarian assistance with the establishment of the Gaza Humanitarian Foundation (102). Yet, even as international humanitarian efforts have been blocked, individuals have sought to keep essential elements functioning; this is especially evident in the efforts of both Palestinian and international medical practitioners and other humanitarian workers (58). They have also sought to resist and disrupt the intent of the Israeli government by publicly sharing their experiences with a global audience and telling the stories of children as mesosystems have been systematically, purposefully, and rapidly dismantled.

The exosystem incorporates those influences within which children and their families operate daily, shaping children's environments even when the children do not interact directly with them. Parent's work and social connections are often included in the exosystem, creating the context for a child's life even though the child does not directly interact with it. Sagi-Schwartz and colleagues have argued that the exosystem can create “safe havens” for children, providing positive interactions of care and support, but also highlight the pressure to which exosystems in Gaza were subjected even before the most recent conflict as a result of Israeli occupation, conflict, and human rights abuses from both Israeli forces and the Hamas leadership (105, 106). This third circle, as Bronfenbrenner (107) characterizes the exosystem, already fragile, has been decimated in Gaza since October 2023 [(63), p. 54]. The services and interactions that facilitate the types of care, education and engagement children experience have been under direct assault.

## A macrosystem on the brink of collapse

Bronfenbrenner [(6), p. 9] characterized the macrosystems “involving generalized patterns of ideology and institutional structure characteristic of a particular culture or subculture”. The macrosystem also includes economic and political systems. For our purposes, we are characterizing the macrosystem as including both the ideology and values described by Bronfenbrenner and the structures that enable the physical enactment of those values. We argue here that the extent of physical destruction means that the values and norms of the macrosystem are reshaped and eroded, with deep implications for children.

As the narrative review demonstrates, infrastructure—a significant manifestation of the physical macrosystems surrounding children—had been destroyed. Eighty percent of critical water, sanitation, hygiene, energy and waste management facilities were destroyed (108). Sixty percent of homes and 65% of roads have been destroyed, with the attacks resulting in more than 50 million tons of debris. Under the debris human remains are buried (A/79/739). In February 2025, the British Medical Journal reported on the destruction of hospitals and health centers, described as the “complete evisceration” of the healthcare system. Beyond the mass deaths resulting directly from the attacks of the Israeli Defense Force, there are uncounted deaths from a lack of treatment and services for illness and disease (58).

The impact of the war on children must be understood within the context of the physical destruction of the macrosystem that is critical to children's lives. Significantly, Bronfenbrenner (6, 9) highlights the importance of public policy in creating the normative preconditions and the legislative framework for children's development and well-being. The destruction wrought on Gaza, leaves little scope for public policy to support and protect children in the face of onslaught in the near future. The extent and depth of destruction mean the macrosystem is on the brink of collapse.

## Trauma over time: the chronosystem

Introduced into his ecological theory in 1986, Bronfenbrenner describes the chronosystem as influencing “the person's development of changes (and continuities) over time in the environments in which the person is living” [(7), p. 724]. The chronosystem links people, events, and all other systems referred to in this article across the axis of time [see (105)]. Sagi-Schwartz (105) has highlighted the ways in which the long history of the Palestinian-Israeli conflict creates a chronosystem within which each conflict plays out. The experience of children in Gaza since October 2023 must be understood within the ongoing conflict that has shaped every element of their environment, and the history of their families and communities.

In 1947, UN Resolution 181 divided the Palestinian territories into two states: one Jewish and one Arab. The plan was rejected by Arab states and set the context for decades of conflict between the Jewish and Arab communities (109). Between late 1947 and early 1949, over 750,000 Palestinians were dispossessed of their land and homes by Zionist paramilitary forces (110). Following the declaration of the state of Israel in 1948, war broke out between Israel and a coalition of Arab nations, and while the war ended in 1949, lasting peace has never been achieved. The displacement of hundreds of thousands of Palestinians during the Nakba (catastrophe) throughout 1948 has been described as “the turning point in the modern history of Palestine … a year of traumatic rupture in the continuity of historical space and time in Palestinian history” [(110), p. 3]. Stefanini [(111), p. 139] makes a crucial point in observing that a “a defining characteristic of the Nakba is that it has never ended.” Those who were displaced, and particularly those who became refugees, have experienced collective and intergenerational trauma (112, 113). Veronese et al. [(113), p. 1815] describe “feelings of helplessness and grief [which] are connected to the historical sense of dispossession and anxiety that Palestinians have experienced for generations”.

Events since the 1940s are embedded within both a history of grief and trauma and a context on ongoing conflict. In the wake of the Nakba, the Gaza Strip became a permanent site for refugee camps, providing ongoing accommodation for many hundreds of thousands of people who had expected to return to their homes within weeks (114). From 1967 war to 2005, the Gaza Strip was occupied by Israeli forces. This period was shaped by dispossession, oppression and structural violence (115, 116), as Gaza became economically dependent on Israel (134). Following the First Intifada, or Palestinian uprising, in 1987, relations between Israel and Gaza deteriorated into open conflict, despite periods characterized by the promise of peace (117). Following the Second Intifada, conflict escalated (115, 116). In 2005, the Israeli government withdrew from Gaza, only to impose a blockade following the Hamas takeover of the territory in 2007. The blockade resulted in serious deterioration in basic services, living standards and the economy in Gaza (117). As a result of the decision of the Israeli government to include essential supplies, including food, in the blockade, 80% of Gazans were reliant on food aid as hunger and malnutrition became widespread (117). Moreover, Mukhimer (106) has documented human rights violations against Gazans under the authority of Hamas. While a detailed account of the history of conflict between Israel and the Occupied Palestinian Territories, or the history of Gaza, is beyond the scope of this article,^1^ the point here is that children impacted by the attacks on Gaza since 2023 have experienced little but violence and conflict throughout their young lives. The current experience of children in Gaza must be understood within the ongoing conflict that has shaped every element of their environment, and the history of their families and communities. The attacks on Gaza between October 2023 and August 2025 created a chronosystem that will reverberate for generations to come.

There is growing evidence that parents' exposure to conflict, war and displacement impacts the health and developmental outcomes of their children and creates intergenerational effects (119). The significance of the chronosystem in understanding the depth of intergenerational conflict and trauma has been powerfully demonstrated in recent research on the ways in which maternal trauma affects the fetus in-utero and into adulthood (15). In one of the first studies of prenatal exposure to war-related violence and conflict across generations, Mulligan and colleagues (15) identified an intergenerational epigenetic signature of trauma in humans. Children experiencing war-related violence in Gaza today likely were affected by the trauma of their mothers in utero, while their experiences as children are likely to be passed on to future generations. This is especially significant given the severity of violence and human rights violations being inflicted on children in Gaza.

## The geopolitical/geostrategic system

Sagi-Schwartz [(105), p. 941] extends Bronfenbrenner's ecological model to include the geopolitical/geostrategic system, described as being distal for any individual, but highly consequential. In the most recent conflict, the geopolitical/geostrategic situation is embedded in the painful history described in regard to the chronosystem, the conflict between Israel and Hamas, the war prosecuted by Israel against Gaza between October 2023 and August 2025, and the geostrategic and political considerations of powerful nation states. A defining feature of the attacks on Gaza since October 2023 is the inaction of international actors. There has been a complete failure to act to end the conflict, to respond to what Jacob (41) has described as “the wider policies enacting the war”, to impose meaningful sanctions, or to place sufficient pressure on Israeli leaders, including those for whom arrest warrants have been issued by the International Criminal Court (120). Even as the International Court of Justice issues provisional measures finding a “real and imminent risk” of genocide occurring in Gaza in the first months of the attacks on Gaza (34), there was little response from political leaders, particularly in established democracies.

Bar-Tal (121) has described the deeply competing narratives that have surrounded the attacks on Israel by Hamas in October 2023, highlighting how those narratives in turn shape the way in which the Israeli government's response has been interpreted. Central to those interpretations is whether the events of the Hamas attacks are considered as a singular event or as part of a broader history of violence and oppression (122). The Hamas attacks, which resulted in 1,200 deaths, egregious violence against civilians, and the taking of over 240 hostages, have been widely condemned (123); and the killing of children by any force is deplorable [see (41)]. McMahan [(88), p. 5] has persuasively argued that while Hamas holds a level of moral responsibility, the horrors that have been inflicted on the civilian population of Gaza, and particularly on children, “are all the result of unnecessary, disproportionate, and indiscriminate warfare for which Israel—meaning the relevantly involved Israelis in the government, military, and so on—is not only responsible but also highly culpable.” Giroux [(91), p. 120] argues the violence perpetrated by Hamas on 7 October 2023, “however horrendous, is not equivalent to the suffering and terror imposed by the Israeli state on Palestinians both historically and in light of the current escalating scale of what amounts to massive, unthinkable, and unconscionable violence.”

While narratives told and ideological positions taken partly explain inaction in the face of egregious violations of children's human rights, as documented in the narrative review here and by multiple sources since October 2023, so too do strategic and economic interests of major powers (124–126). It is beyond the scope of this article to analyse the ways in which strategic and economic interests have contributed to international inaction in the face of actions that are increasingly being described as genocide (34, 46, 127). As noted earlier, the world has been complicit through the failure to act (77). However, some countries have enabled Israeli military efforts, not only through the failure to apply sanctions but by continuing to actively engage in the trade of weaponry with Israel. El-Shewy and colleagues (125) have documented the extent to which the highly-profitable global military-industrial complex, supported by global powers, enables Israel's military capability. Additionally, principles of human rights and humanitarianism have been replaced by the militarisation of humanitarian assistance through the Gaza Humanitarian Foundation, which has been actively supported by the United States (128). In August 2025, UN experts issued the following statement:

The GHF, a non-governmental organization created by Israel in February 2025, with US support, to allegedly distribute aid in Gaza, is an utterly disturbing example of how humanitarian relief can be exploited for covert military and geopolitical agendas in serious breach of international law (138).

The Gaza Humanitarian Foundation is the embodiment of strategic and economic interests converging to capitalize on the suffering of the people of Gaza. Both the GHF and the maintenance of the international weapons trade with Israel highlight the extent to which the geopolitical/geostrategic system has deepened the human catastrophe that has played out in Gaza.

## Discussion

**Figure 2:**
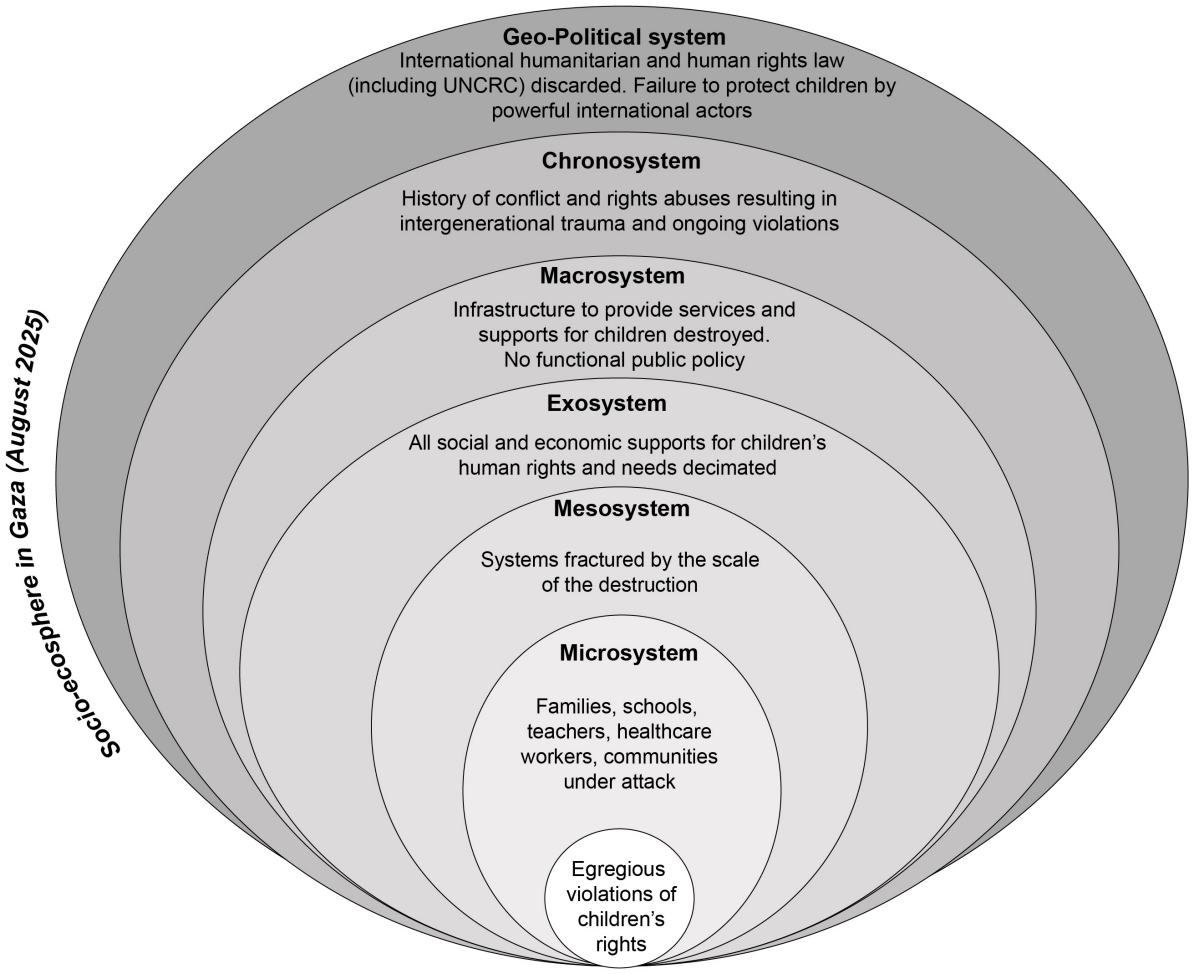
Children's socio-ecosphere in Gaza in August 2025.

The socio-ecosphere in Gaza is characterized by egregious violations of children's human rights and the denial of fundamental needs, resulting from the dismantling of every system that has the potential to support children. Within the geo-political system, international law has been discarded as powerful actors have failed to uphold children's human rights. Analysis of the chronosystem demonstrates the depth of intergenerational trauma and the ongoing human rights violations to which the current generation of Gazan children have been subjected. The macro-system was not respecting of children's human rights prior to Israel's war on Gaza following the Hamas attacks of 7 October 2023, but the assaults from October 2023 to August 2025 has demolished the infrastructure within which the macro-system is embedded. Moreover, the assaults on Gaza have made the policies essential for the delivery of services impossible. The economic and social supports for children's human rights and basic needs that characterize the exosystem have been decimated. Linkages between systems have been fractured irreparably as a result of the scale of the destruction. Families, schools and teachers, healthcare workers and communities—the essential microsystem that structures children's everyday lives—have been attacked to the point of breakdown. The socio-ecosphere as it now exists in Gaza, and the relationship between the destruction of ecosystems and the violation of children's human rights, is represented in Figure 2.

The horrific violations of children's rights cataloged in the narrative review have devastating results for individual children. Yet, however egregious individual violations are, it is arguably the destruction of every socio-ecological system within the socio-ecosphere that threatens the collective identity, culture and right to survival of Palestinian children in Gaza. It is the reconstruction of the socio-ecosphere that is now both the challenge and responsibility of an international community that failed to act as the violation of children's human rights unfolded.

**Figure 3:**
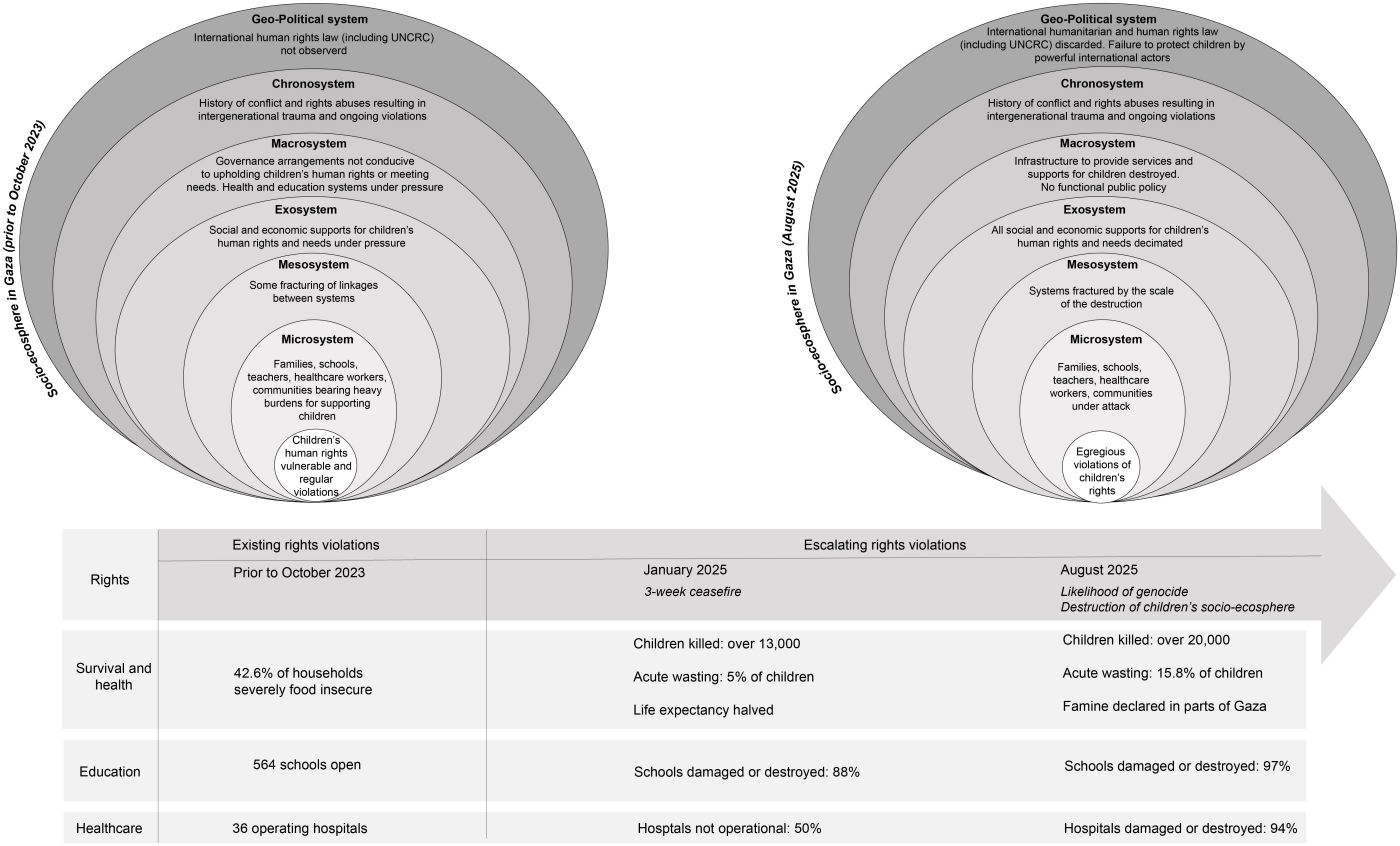
The dismantling of children's socio-ecosphere.

It is important to recognize that prior to October 2023, the socio-ecosphere in Gaza did not reflect a rights-respecting and child inclusive society, able to uphold children's human rights and meet their needs. Children's basic needs were met and there were widespread rights violations. Yet, as our analysis here demonstrates, during the period October 2023 to August 2025, the socio-ecosphere that shapes the socio-ecosystems within which children live has been effectively annihilated. Figure 3 represents this process.

## Conclusion

The assault on Gaza since October 2023 signals an historic moment. There is well-established literature on the ways in which conflict shapes children's lives and creates ongoing trauma. There is a well-established international system of humanitarian and human rights law that emphasis the protection of children (and civilians generally). Since Graca Machel's report of the *Impact of Armed Conflict on Children* in 1996, global rhetoric around the protection of children in war has intensified. None of this evidence has served to protect children in Gaza or mitigate the assaults on them since October 2023.

As documented here, violations of children's human rights have been egregious not only in scale but in intensity and in the targeting of children. The nature of those violations is such that children's socio-ecosphere has been systematically dismantled. Damage to microsystems, mesosystems, exosystems and marcosystems indicate the ways in which every dimension of children's lives have been under assault, violating children's human rights and most basic needs directly, and striping away protective factors. By introducing the chronosystem as a foundational level of children's ecosystems, we have shown the intergenerational nature of trauma already experienced by children in Gaza. The attacks not only reinforce existing intergenerational trauma but create future trauma that will shape the lives of generations to come. As Taylor has argued, conflicts that evolve over generations need generations to heal (129).

The narrative review provided here not only synthesizes the violations inflicted on children but demonstrates the evidence available. Killings, maiming, denial of healthcare and education, and starvation have all played out in full view, and with full knowledge of the world. Inaction to halt violations of children's human rights and the destruction of the ecosystem that shapes their lives and futures is not due to a lack of knowledge, as the August 2025 declaration of the International Genocide Studies Association makes clear (5). As we discuss, the geopolitical/geostrategic systems at play help to explain inaction but can never justify it.

El-Afendi [(127), p. 7] has questioned the future of genocide studies after Gaza, arguing:

One maxim it should state is: if a series of actions approach genocide sufficiently to occasion a debate on whether they are genocide or not, then they are evil enough to be denounced without ifs or buts; even more so if the aim is to sustain an unjust system. There is something seriously wrong if there are too many influential actors prevaricating on this.

We raise similar questions about the future of children's human rights and the protection of children during war and conflict. That powerful global actors have not acted despite the evidence of gross human rights violations and, increasingly, of genocide is devastating for children in Gaza today and for future generations. It may also be devastating for any child impacted by war or conflict anywhere. As in the immediate World War Two period, the world is facing a pivotal moment in international commitment to peace and justice. Whatever occurs next, the lives of hundreds of thousands of children in Gaza have been destroyed as the world watched a moral, human rights and public health crisis unfold. The least to be demanded of global actors now is that they commit to rebuilding the lives and the worlds of children in Gaza, and act immediately to protect their right to identity by preventing further actions to drive Palestinians from Gaza.

## Data Availability

The original contributions presented in the study are included in the article/supplementary material, further inquiries can be directed to the corresponding author.

## Footnotes

1 See Caplan (118) for a useful analysis of the conflict.

## References

[1] United Nations. Gaza “Becoming a Graveyard for Children”, Warns UN Secretary-General, Calling for Humanitarian Ceasefire. Press Release. SM/SM/2202 (2023). Available online at: https://www.un.org/unispal/document/gaza-becoming-a-graveyard-for-children-warns-un-secretary-general-calling-for-humanitarian-ceasefire-press-release/ (Accessed January 9, 2025).

[2] Independent International Commission of Inquiry on the Occupied Palestinian Territory including East Jerusalem and Israel. Legal Analysis of the Conduct of Israel in Gaza Pursuant to the Convention on the Prevention and Punishment of the Crime of Genocide. Human Rights Council (2025).

[3] Byman D. A war they both are losing: Israel, Hamas and the Plight of Gaza. Survival. (2024) 66:61–78. doi: 10.1080/00396338.2024.2357484

[4] Fennig M Snir A Shorer M Harlev EB Fennig S. Early psychological responses of children and caregivers in the immediate aftermath of release from war captivity. Front Psychol. (2025) 16:1588422. doi: 10.3389/fpsyg.2025.1588422 40486893 PMC12141228

[5] IAGS. International Association of Genocide Studies Resolution on the Situation in Gaza. Johannesburg: IAGS (2025).

[6] Bronfenbrenner U. The Ecology of Human Development: Experiments by Nature and Design. Cambridge, MA: Harvard University Press (1979). doi: 10.4159/9780674028845

[7] Bronfenbrenner U. Ecology of the family as a context for human development: research perspectives. Dev Psychol. (1986) 22:723–42. doi: 10.1037//0012-1649.22.6.723

[8] Bronfenbrenner U. Ecological model of human development. In:Postlethwaite TN Husen T, editors. International Encyclopedia of Education. Oxford: Elsevier (1994).

[9] Ladd GW Cairns E. Children: ethnic and political violence. Child Dev. (1996) 67:14–8. doi: 10.1111/j.1467-8624.1996.tb01715.x 8605824

[10] UN Secretary General. Children and Armed Conflict. Report Submitted to the General Assembly Pursuant to Resolution 78/187. A78/842. United Nations (2024).

[11] UN Secretary General. Children and Armed Conflict. Report Submitted to the General Assembly Pursuant to Resolution 78/187. A/79/878. United Nations (2025).

[12] Barenbaum J Ruchkin V Schwab-Stone M. The psychosocial aspects of children exposed to war: practice and policy initiatives. J Child Psychol Psychiatry. (2004) 45:41–62. doi: 10.1046/j.0021-9630.2003.00304.x 14959802

[13] Hazer L Gredebäck G. The effects of war, displacement, and trauma on child development. Humanit Soc Sci Commun. (2023) 10:909. doi: 10.1057/s41599-023-02438-8

[14] Manduca P Rothchild A Meyers A Tognoni G Summerfield D Balduzzi A . On the duty to protect the people of Gaza: how the collapse of the hospital health care system has reinforced genocidal intent. J Public Health Emerg. (2024) 8. doi: 10.21037/jphe-24-11

[15] Mulligan CJ Quinn EB Hamadmad D Dutton CL Nevell L Binder AM . Epigenetic signatures of intergenerational exposure to violence in three generations of Syrian refugees. Sci Rep. (2025) 15:5945. doi: 10.1038/s41598-025-89818-z 40016245 PMC11868390

[16] Manzanero AL Crespo M Barón S Scott T El-Astal S Hemaid F. Traumatic events exposure and psychological trauma in children victims of war in the Gaza strip. J Interpers Violence. (2021) 36:1568–87. doi: 10.1177/0886260517742911 29294997

[17] Abudayya A Bruaset GTF Nyhus HB Aburukba R Tofthagen R. Consequences of war-related traumatic stress among Palestinian young people in the Gaza Strip: a scoping review. Ment Health Prev. (2023) 32:200305. doi: 10.1016/j.mhp.2023.200305

[18] Feldman R Vengrober A. Posttraumatic stress disorder in infants and young children exposed to war-related trauma. J Am Acad Child Adolesc Psychiatry. (2011) 50:645–58. doi: 10.1016/j.jaac.2011.03.001 21703492

[19] Green AH Kocijan-Hercigonja D. Stress and coping in children traumatized by war. J Am Acad Psychoanal. (1998) 26:585–97. doi: 10.1521/jaap.1.1998.26.4.585 10096056

[20] Williams R. The psychosocial consequences for children of mass violence, terrorism and disasters. Int Rev Psychiatry. (2007) 19:263–77. doi: 10.1080/09540260701349480 17566904

[21] Begovac I Rudan V Begovac B Vidović V Majić G. Self-image, war psychotrauma and refugee status in adolescents. Eur Child Adolesc Psychiatry. (2004) 13:381–8. doi: 10.1007/s00787-004-0423-x 15619051

[22] Berman H. Children and war: current understandings and future directions. Public Health Nurs. (2001) 18:243–52. doi: 10.1046/j.1525-1446.2001.00243.x 11468064

[23] Macksoud MS Aber JL. The war experiences and Psychosocial development of children in Lebanon. Child Dev. (1996) 67:70–88. doi: 10.1111/j.1467-8624.1996.tb01720.x 8605835

[24] Stevanović A Frančišković T Vermetten E. Relationship of early-life trauma, war-related trauma, personality traits, and PTSD symptom severity: a retrospective study on female civilian victims of war. Eur J Psychotraumatol. (2016) 7:30964. doi: 10.3402/ejpt.v7.30964 27056034 PMC4824847

[25] Alsalem R. Sex-Based Violence Against Women and Girls: New Frontiers and Emerging Issues: Report of the Special Rapporteur on Violence Against Women and Girls, Its Causes and Consequences (2025).

[26] Chasson M Ben-Shlomo S Lyons-Ruth K. Early parent–child relationship in the shadow of war-related trauma: a systematic review. Trauma Violence Abuse. (2025). doi: 10.1177/15248380251325222 40099528

[27] Akbulut-Yuksel M. Children of war. J Hum Resour. (2014) 49:634–62. doi: 10.1353/jhr.2014.0021

[28] Pape RA. The Unparalleled Devastation of Gaza: Why Punishing Civilians Has Not Yielded Strategic Success. Foreign Affairs (2025). [WWW]. Available online at: https://www.foreignaffairs.com/israel/gaza-unparalleled-devastation-robert-pape?utm_medium=promo_emailandutm_source=lo_flowsandutm_campaign=article_linkandutm_term=article_emailandutm_content=20250908 (Accessed September 8, 2025).

[29] International Committee of the Red Cross. The Geneva Conventions of 12 August 1949. Geneva: ICRC (1949).

[30] Bellal A Casey-Maslen S. The protection of children. In: The Additional Protocols to the Geneva Conventions in Context. Oxford: Oxford University Press (2022). p. 51–67. doi: 10.1093/law/9780192868909.003.0005

[31] ICRC. Additional Protocols to the Geneva Conventions of 1949. Geneva: International Committee of the Red Cross (1977).

[32] Dixit RK. Special protection of children during armed conflicts under the Geneva conventions regime. In: ISIL Year Book of International Humanitarian and Refugee Law 9th, ed. New Delhi: ISIL (2001).

[33] McGreal. ‘Not a Normal War': Doctors Say children Have Been Targeted by Israeli Snipers in Gaza. Gaza: The Guardian (2024). Available online at: https://www.theguardian.com/world/2024/apr/02/gaza-palestinian-children-killed-idf-israel-war (Accessed August 22, 2025).

[34] University Network for Human Rights. Genocide in Gaza: Analysis of International Law and its Application to Israel's Military Actions Since October 7, 2023 (2024). Available online at: https://www.humanrightsnetwork.org/publications/genocide-in-gaza (Accessed August 23, 2025).

[35] Azoulay A. Palestine as symptom, Palestine as hope: revising human rights discourse. Crit Inq. (2014) 40:332–64. doi: 10.1086/676411

[36] Witt JF. The dismal history of the laws of war. UC Irvine L Rev. (2011) 895–912.

[37] Wessells MG. The changing nature of armed conflict and its implications for children: the Graca Machel/UN Study. J Peace Psychol. (1998) 4:321–34. doi: 10.1207/s15327949pac0404_2

[38] Lee-Koo K. “The intolerable impact of armed conflict on children”: The United Nations security council and the protection of children in armed conflict. GRP. (2018) 10:57–74. doi: 10.1163/1875984X-01001004

[39] Al-Najjar A Zaid B. Western media's ethical collapse: silencing Gaza's voice. Third World Q. (2025) 1–20. doi: 10.1080/01436597.2025.2552361

[40] Abrams E. UN Halves Its Estimate of Women and Children Killed in Gaza | Council on Foreign Relations. Washington, DC: Council on Foreign Relations (2024).

[41] Jacob C. Prosecuting international crimes against children: prospects for intergenerational justice in international security. Crit Stud Secur. (2025) 13:270–3. doi: 10.1080/21624887.2025.2502721

[42] Jacob C. A new politics of international criminal justice: accountability in Ukraine and the Israel-Gaza war. Int Aff. (2024) 100:2563–81. doi: 10.1093/ia/iiae224

[43] Jane Esparrago-Kalidas A. The effectiveness of CRAAP test in evaluating credibility of sources. Int J TESOL Educ. (2021) 1:1–14.

[44] Blakeslee S. The CRAAP test. LOEX Q. (2004) 31:4.

[45] OHCHR DPA DPKO. Public Reporting on Human Rights by United Nations Peace Operations: Good Practices, Lessons Learned and Challenges (2017). [WWW Document]. Available online at: https://resourcehub01.blob.core.windows.net/$web/Policy and Guidance/corepeacekeepingguidance/Thematic Operational Activities/Human Rights/Public Reporting on Human Rights by United Nations Peace Operations Good Practices, Lessons Learned and Challenges.pdf (Accessed September 8, 2025).

[46] Malhotra R El-Shaarawi N. We want to live as other children live': Palestinian children's resistance to genocide as ‘fighting for life. Gend Place Cult. (2025) 32:1–14. doi: 10.1080/0966369X.2025.2484683

[47] Liu M. War and children. Am J Psychiatry Resid J. (2017) 12:3–5. doi: 10.1176/appi.ajp-rj.2017.120702

[48] Mandalakas AM. The greatest impact of war and conflict. Ambul Child Health. (2001) 7:97–103. doi: 10.1046/j.1467-0658.2001.0115a.x

[49] Santa Barbara J. Impact of war on children and imperative to end war. Croat Med J. (2006) 47:891–4. 17167852 PMC2080482

[50] Boukari Y Kadir A Waterston T Jarrett P Harkensee C Dexter E. Gaza, armed conflict and child health. BMJ Paediatr Open. (2024) 8:e002407. doi: 10.1136/bmjpo-2023-002407 38350977 PMC10868171

[51] El Kishawi RR Soo KL Abed YA Muda WAMW. Prevalence and associated factors influencing stunting in children aged 2-5years in the Gaza Strip-Palestine: a cross-sectional study. BMC Pediatr. (2017) 17:210. doi: 10.1186/s12887-017-0957-y 29268788 PMC5740756

[52] Massad SG Nieto FJ Palta M Smith M Clark R Thabet AA. Health-related quality of life of Palestinian preschoolers in the Gaza Strip: a cross-sectional study. BMC Public Health. (2011) 11:253. doi: 10.1186/1471-2458-11-253 21510877 PMC3094247

[53] Waterston T Nasser D. Access to healthcare for children in Palestine. BMJ Paediatr Open. (2017) 1:e000115. doi: 10.1136/bmjpo-2017-000115 29637139 PMC5862189

[54] Al Shami A Nashwan AJ. Challenges of children amputees in Gaza. East Mediterr Health J. (2025) 31:233–4. doi: 10.26719/2025.31.4.233 40448486

[55] Mahase E. Gaza-Israel conflict: hundreds of medics are killed or arrested after intense attacks on healthcare facilities. BMJ. (2024) 384:q203. doi: 10.1136/bmj.q203 38272530

[56] Liddell G. Palestinian Writers Have Long Explored the Horrors of Amputation. London: The Conversation (2024). doi: 10.64628/AAI.h6d43nmxu

[57] UNICEF. A Year of Tears: 12 Months of War on Children - Situation Analysis (2024). Available online at: https://www.unicef.org/sop/media/4566/file/One%20year%20of%20Gaza%20Escalation.pdf.pdf (Accessed September 1, 2025).

[58] Mahase E. Gaza's health system is “completely eviscerated” - what happens now? Br Med J. (2025) 388: r361. doi: 10.1136/bmj.r361 40000069

[59] World Health Organization. Health System at Breaking Point as Hostilities Further Intensify in Gaza. [WWW Document]. Geneva: World Health Organization (2025).

[60] Irfan B Lulu I Hamawy A Shammala AA Kullab S Fawaz M . combating infections under siege: healthcare challenges amidst the military assault in Gaza. World Med Health Policy. (2025) 17:188–213. doi: 10.1002/wmh3.642

[61] Shellah D. War on Gaza: the impossible duty to care for the critically ill. Intensive Care Med. (2024) 50:311–3. doi: 10.1007/s00134-023-07309-z 38252287

[62] Harghandiwal B. Impact of the humanitarian crisis in Gaza on children's health: evidence and recommendations for mitigation. Glob Public Health. (2025) 20:2495326. doi: 10.1080/17441692.2025.2495326 40260702

[63] UNICEF. Humanitarian Situation Report No. 40 (2025). Available online at: https://www.unicef.org/documents/state-palestine-humanitarian-situation-report-no-40-01-january-30-june-2025-mid-year (Accessed August 29, 2025).

[64] UNICEF. Ten Weeks of Hell' for Children in Gaza (2023). Available online at: https://palestine.un.org/en/256251-'ten-weeks-hell'-children-gaza-unicef (Accessed September 2, 2025).

[65] Grotto I Agha H Al-Halaweh A Davidovitch N MdKee M. Public health, war and cross-border challenges: the recent cVDPV2 polio outbreak in Gaza. EClinicalMedicine. (2025) 81:103–36. doi: 10.1016/j.eclinm.2025.103136 40104084 PMC11919384

[66] Kunichoff D Mills D Asi Y Abdulrahim S Wispelwey B Tanous O. Are hospitals collateral damage? Assessing geospatial proximity of 2000 lb bomb detonations to hospital facilities in the Gaza Strip from October 7 to November 17, 2023. PLOS Global Public Health (2024) 4:e0003178. doi: 10.1371/journal.pgph.0003178 39387878 PMC11466298

[67] Human Rights Watch. Israel: White Phosphorus Used in Gaza, Lebanon (2023). [WWW Document]. Available online at: https://www.hrw.org/news/2023/10/12/israel-white-phosphorus-used-gaza-lebanon (Accessed September 7, 2025).

[68] Rosen R Moghli M. Israel's war on Gaza is deliberately targeting children – New UN Report (2025). doi: 10.64628/AB.34n447yxc

[69] Galari I. Opinion: I'm an American doctor who went to Gaza. I saw annihilation, not war (2024). Los Angeles Times. Available online at: https://www.latimes.com/opinion/story/2024-02-16/rafah-gaza-hospitals-surgery-israel-bombing-ground-offensive-children (Accessed September 7, 2025).

[70] Palestinian Return Centre. Written Statement Submitted by The Palestinian Return Centre Ltd, a Non-Governmental Organization in Special Consultative Status (2024).

[71] Hassoun A Jarrar H Goudali L Lisciani S Al-Muhannadi K Buheji M . Life on land in peril: how the recent war on gaza has devastated terrestrial ecosystems. In:Hassoun A., editors, *War on Gaza. Sustainable Development Goals Series*. Cham: Springer (2025). doi: 10.1007/978-3-031-88500-6_13

[72] Abuawad A Griffiths M Edwards GH Eftekhari A Al-Ebweini M Al-Najar H . The ongoing environmental destruction and degradation of Gaza: the resulting public health crisis. Am J Public Health. (2025) 115:1053–61. doi: 10.2105/AJPH.2025.308140 40499107 PMC12160645

[73] Ganguly M. ‘A Deadly Scheme': Palestinians Face Indiscriminate Gunfire at Food Sites. Gaza: The Guardian (2025). Available online at: https://www.theguardian.com/world/ng-interactive/2025/aug/09/a-deadly-scheme-palestinians-face-indiscriminate-gunfire-at-food-sites (Accessed September 1, 2025).

[74] Haan N Hailey P Maxwell D Seal A Lopez J Russo L . Famine Review Committee: Gaza Strip, August. Rome: Integrated Food Security Phase Classification (IPC) Global Initiative (2025).

[75] Vassilakou T. Childhood malnutrition: time for action. Children. (2021) 8:103. doi: 10.3390/children8020103 33546298 PMC7913494

[76] Martin S Warner JG Fagen P John G. Palestinian Refugees in Gaza. Fordham Int Law J. (2005) 28:1457–8.

[77] OHCHR. UN Experts Declare Famine has Spread Throughout Gaza Strip (2024). [WWW Document]. United Nations Office of the High Commissioner for Human Rights. Available online at: https://www.ohchr.org/en/press-releases/2024/07/un-experts-declare-famine-has-spread-throughout-gaza-strip (Accessed August 19, 2025).

[78] World Food Program. Gaza Conflict: A Timeline of Aid in a Humanitarian Crisis (2024). [WWW Document]. World Food Program. Available online at: https://www.wfpusa.org/news/gaza-conflict-timeline-aid-humanitarian-crisis/ (Accessed August 19, 2025).

[79] Alflah YM Alrashidi M. Severe Acute Malnutrition and Its Consequences Among Malnourished Children. J Clin Pediatr Res. (2023) 2. doi: 10.37191/Mapsci-2583-4525-2(1)-011

[80] Picot J Hartwell D Harris P Mendes D Clegg AJ Takeda A. The effectiveness of interventions to treat severe acute malnutrition in young children: a systematic review. Health Technol Assess. (2012) 16:1–316. doi: 10.3310/hta16190 22480797 PMC4781582

[81] Ververs M. Identification of Acute Malnutrition, Adverse Birth Outcomes and Nutritional Care for Pregnant, Lactating Women in Emergencies or Protracted Crises. Geneva: Médecins Sans Frontières (2011).

[82] Aguilar P Retamal G. Protective environments and quality education in humanitarian contexts. Int J Educ Dev. (2009) 29:3–16. doi: 10.1016/j.ijedudev.2008.02.002

[83] Salha S Tlili A Shehata B Zhang X Endris A Arar K . How to maintain education during wars? An integrative approach to ensure the right to education. Open Praxis. (2024) 16:160–79. doi: 10.55982/openpraxis.16.2.668

[84] OCHA. Hostilities in the Gaza Strip and Israel | Flash Update #25. [WWW Document] (2023). Available online at: https://www.ochaopt.org/content/hostilities-gaza-strip-and-israel-flash-update-25 (Accessed August 9, 2025).

[85] Faculty of Education, University of Cambridge, Centre for Lebanese Studies and UNRWA. Palestinian Education Under Attack in Gaza: Restoration, Recovery, *Rights and Responsibilities in and Through Education*. Cambridge: Faculty of Education, University of Cambridge (2024).

[86] Relief Web. Education Overview in 2024: The State of Education in Gaza and the West Bank “Current Realities and Future Priorities” (2025). [WWW Document]. Available online at: https://reliefweb.int/report/occupied-palestinian-territory/education-overview-2024-state-education-gaza-and-west-bank-current-realities-and-future-priorities-february-2025 (Accessed September 7, 2025).

[87] Moshman D. Us and them: identity and genocide. Identity. (2007) 7:115–35. doi: 10.1080/15283480701326034

[88] McMahan J. Moral responsibility for the scarcity of healthcare in Gaza. J Med Ethics. (2025) 27:jme-2025-111165. doi: 10.1136/jme-2025-111165 40866251

[89] OCHA. Gaza: Children Under Attack (2024). OCHA. [WWW Document]. Available online at: https://www.unocha.org/news/gaza-children-under-attack (Accessed September 7, 2025).

[90] UNICEF. Identity bracelets help keep Gaza Strip children with their families in the midst of the war | UNICEF State of Palestine (2024). [WWW Document]. Available online at: https://www.unicef.org/sop/stories/identity-bracelets-help-keep-gaza-strip-children-their-families-midst-war (Accessed September 7, 2025).

[91] Giroux HA. Genocide in Gaza and the Politics of False Equivalencies. Policy Pract A Dev Educ Rev. (2024) 38:120–5.

[92] Independent International Commission of Inquiry on the Occupied Palestinian Territory including East Jerusalem and Israel. “More than a human can bear”: Israel's systematic use of sexual, reproductive and other forms of gender-based violence since 7 October 2023. Report to fifty-eigth session of the Human Rights Council. A/HRC/58/CRP.6 (2025).

[93] OHCHR. End Unfolding Genocide or Watch It End Life in Gaza: UN Experts Say States Face Defining Choice (2025). [WWW Document]. Available online at: https://www.ohchr.org/en/press-releases/2025/05/end-unfolding-genocide-or-watch-it-end-life-gaza-un-experts-say-states-face (Accessed August 19, 2025).

[94] United Nations Relief Works and Agency. UNRWA Situation Report #182 on the Humanitarian Crisis in the Gaza Strip and the West Bank, including East Jerusalem, Friday, August 1, 2025 (2025). [WWW Document]. Available online at: https://www.unrwa.org/resources/reports/unrwa-situation-report-182-situation-gaza-strip-and-west-bank-including-east-jerusalem (Accessed September 9, 2025).

[95] Chetail V. Removing Palestinians from Gaza is not a plan; it is a crime against humanity. EJIL. (2025).

[96] Gal T. A socioecological model of children's rights. In:Todres J King SM., editors, *The Oxford Handbook of Children's Rights Law*. Oxford: Oxford University Press (2020). pp. 118–37. doi: 10.1093/oxfordhb/9780190097608.013.7

[97] Kiyada S Scarr S Al-Mughrabi N. The Lives Lost in Gaza: A Closer Look at Those Killed in the Conflict so Far (2025). Reuters. [WWW Document]. Available online at: https://www.reuters.com/graphics/ISRAEL-PALESTINIANS/FATALITIES/byvrxlqeqve/ (Accessed July 9, 2025).

[98] Rosa EM Tudge J. Urie Bronfenbrenner's theory of human development: its evolution from ecology to bioecology. J Fam Theory Rev. (2013) 5:243–58. doi: 10.1111/jftr.12022

[99] Diab SY Palosaari E Punamäki RL. Society, individual, family, and school factors contributing to child mental health in war: the ecological-theory perspective. Child Abuse Neglect. (2018) 84:205–16. doi: 10.1016/j.chiabu.2018.07.033 30118970

[100] Qouta S Odeh J. The impact of conflict on children: the Palestinian experience. J Ambul Care Manage. (2004) 28:75–9. doi: 10.1097/00004479-200501000-00010 15682964

[101] Rabaia Y Kassis S Amro Z Giacaman R Reis R. Coping and helping to cope: perspectives of children of Palestinian political detainees. Child Soc. (2018) 32:345–56. doi: 10.1111/chso.12263

[102] United Nations. UN experts call for immediate dismantling of Gaza Humanitarian Foundation, United Nations Press Release (2025). Available online at: https://www.ohchr.org/en/press-releases/2025/08/un-experts-call-immediate-dismantling-gaza-humanitarian-foundation (Accessed December 30, 2025).

[103] Human Right Watch. Gaza: Israelis Attacking Known Aid Worker Locations (2024). [WWW Document]. Available online at: https://www.hrw.org/news/2024/05/14/gaza-israelis-attacking-known-aid-worker-locations (Accessed August 9, 2025).

[104] World Health Organization. People in Gaza Starving, Sick and Dying as Aid Blockade Continues. [WWW Document]. Geneva: World Health Organization (2025).

[105] Sagi-Schwartz A. Children of war and peace: a human development perspective. J Confl Resolut. (2012) 56:933–51. doi: 10.1177/0022002712446128

[106] Mukhimer T. Hamas Rule in Gaza: Human Rights under Constraint. Berlin: Springer (2013). doi: 10.1057/9781137310194

[107] Bronfenbrenner U. Toward an experimental ecology of human development. Am Psychol. (1977) 32:513–31. doi: 10.1037//0003-066X.32.7.513

[108] OCHA. We Inform (2025). [WWW Document]. OCHA. Available online at: https://www.unocha.org/we-inform (Accessed August 9, 2025).

[109] Center for Preventative Action. Israeli-Palestinian Conflict. Council on Foreign Relations: Global Conflict Tracker (2025).

[110] Masalha N. The Palestine Nakba: Decolonising History, Narrating the Subaltern, Reclaiming Memory. London: Zeb Book (2012).

[111] Stefanini P. Settler-humanitarianism: dispossession, ‘humanitarian transfer' and the 1948 Nakba. Millenn J Int Stud. (2024) 53:136–62. doi: 10.1177/03058298241289371

[112] Ghnadre-Naser S Somer E. “The wound is still open”: the Nakba experience among internally displaced Palestinians in Israel. Int J Migrat Health Soc Care. (2016) 12:238–51. doi: 10.1108/IJMHSC-07-2015-0024

[113] Veronese G Mahamid F Bdier D. Transgenerational trauma and collective resilience: a qualitative analysis of the experiences of settler-colonial violence among three generations of Palestinian refugees. Int J Soc Psychiatry. (2023) 69:1814–24. doi: 10.1177/00207640231175787 37283084

[114] Marx E. Palestinian refugee camps in the West Bank and the Gaza strip. Middle East Stud. (1992) 28:281–94. doi: 10.1080/00263209208700901

[115] Brockhill A Cordell K. The violence of culture: the legitimation of the Israeli occupation of Palestine. Third World Q. (2019) 40:981–98. doi: 10.1080/01436597.2019.1581057

[116] James C. Mere words: the “Enemy Entity” designation of the Gaza strip. Hastings Int Comp Law Rev. (2009) 32:643–68.

[117] Butt KM Butt AA. Blockade on Gaza Strip: A Living Hell on Earth. Blockade on Gaza Strip: A Living Hell on Earth. (2016) 23:157–82.

[118] Caplan N. The Israel-Palestine Conflict: Contested Histories. Maiden: Wiley-Blackwell (2009).

[119] Devakumar D Birch M Osrin D Sondorp E Wells JC. The intergenerational effects of war on the health of children. BMC Med. (2014) 12:57. doi: 10.1186/1741-7015-12-57 24694212 PMC3997818

[120] Soni S. The ICC takes action: the Gaza arrest warrants and the international obligation of states. S Afr J Bioeth Law. (2024) 18:1. doi: 10.7196/SAJBL.2024.v18i1.28741

[121] Bar-Tal D. The told story of the Gaza war. World Affairs. (2025) 188:e70005. doi: 10.1002/waf2.70005

[122] Fassin D. Moral Abdication: How the World Failed to Stop the Destruction of Gaza. Brooklyn, NY: Verso Press (2025).

[123] UN Watch. UN Reactions to Hamas Massacre and Its Aftermath (2023). [WWW Document] Available online at: https://unwatch.org/un-officials-and-bodies-react-to-october-2023-hamas-massacre-and-its-aftermath/ (Accessed August 9, 2025).

[124] Bshara K. Settler colonialism rebranded: Trump's Gaza plan and the capitalist logic of war. J Palest Stud. (2025) 54:62–70. doi: 10.1080/0377919X.2025.2489264

[125] El-Shewy M Griffiths M Jones C. Israel's war on Gaza in a global frame. Antipode. (2025) 57:75–95. doi: 10.1111/anti.13094

[126] Ziadah R. Development as erasure: Palestine, genocide and ‘reconstruction'. Dev Change. (2025) 56:1–22. doi: 10.1111/dech.70014

[127] El-Affendi A. The futility of genocide studies after Gaza. J Genocide Res. (2024) 1–7. doi: 10.1080/14623528.2024.2305525

[128] Hamamra B Shehab E. Humanitarian aid in Gaza: from lifeline to arena of violence and exclusion. Third World Q. (2025) 1–17. doi: 10.1080/01436597.2025.2576787

[129] Taylor LK. The Developmental Peacebuilding Model (DPM) of children's prosocial behaviors in settings of intergroup conflict. Child Dev Perspect. (2020) 14:127–34. doi: 10.1111/cdep.12377

[130] OHCHR. Israel has Committed Genocide in the Gaza Strip, UN Commission Finds. United Nations Press Release (2025). Available online at: https://www.ohchr.org/en/press-releases/2025/09/israel-has-committed-genocide-gaza-strip-un-commission-finds (Accessed December 24, 2025).

[131] Amnesty International. Israel/OPT: US-Made Munitions Used in Two Israeli Airstrikes in Gaza Killed 43 Civilians. Press Release (2023). Available online at: https://www.amnesty.org.uk/press-releases/israelopt-us-made-munitions-used-two-israeli-airstrikes-gaza-killed-43-civilians (Accessed December 24, 2025).

[132] Haan N., Hailey P, Maxwell D, Seal A, Lopez J, Russo L, et al. Famine Review Committee: Gaza Strip, August. Rome: Integrated Food Security Phase Classification (IPC) Global Initiative.

[133] Beaumont P. Israel issues forced displacement orders amid fears of full occupation in Gaza. The Guardian (2025). Available online at: https://www.theguardian.com/world/2025/aug/06/israel-issues-forced-displacement-orders-amid-fears-of-full-occupation-in-gaza (Accessed December 24, 2025).

[134] Winter Y. The siege of Gaza: spatial violence, humanitarian strategies, and the biopolitics of punishment. Constellations. (2016) 6:308–19. doi: 10.1111/1467-8675.12185

[135] United Nations. Convention on the Prevention and Punishment of the Crime of Genocide, opened for signature 9 December 1948, 78. (1948).

[136] Machel G. Machel Study: Impact of Armed Conflict on Children, A/51/306, UN General Assembly, 55th session, Item 108. (1996).

[137] United Nations. Convention on the Rights of the Child. Opened for signature November 20, 1989, General Assembly Resolution 44/25 (entered force 2 September 1990), art 49. New York, NY: United Nations (1989).

[138] UN Experts. UNTS 277 (entered into force 12 January 1951). (n.d.).

